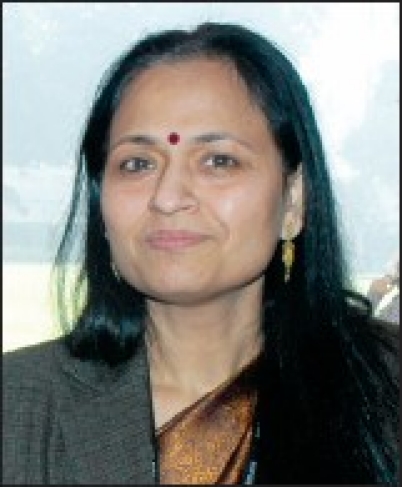# A New Beginning to Excel

**DOI:** 10.4103/0973-1075.53484

**Published:** 2009

**Authors:** Sushma Bhatnagar

**Affiliations:** Chief Editor, IJPC, Associate Professor & Head, Unit of Anesthesiology, Dr. B.R A Institute Rotary Cancer Hospital, All India Institute of Medical Sciences, New Delhi - 110 029, India

It gives me immense pleasure to write as the Chief Editor of the Indian Journal of Palliative Care for the first issue after taking over this responsibility from my predecessor Dr. Joseph Alexander who worked hard to maintain the continuity of this journal despite several constraints and a year of recession. I sincerely thank him for his support and smooth handover.

It is a well-known fact that there are barriers and difficulties to conduct research in the area of palliative care due to reservation and concerns about ethics and clinical trials on patients nearing the end of their life. This constraint makes research in this niche area more challenging and despite all reservations it is highly desirable to contribute authentic evidence-based treatment developments to the scientific knowledge pool. Among others, evidence-based symptom palliation and evolving psychological support mechanism is one of the most important areas in delivering high-quality palliative care.

I wish this journal to become one of the most comprehensive, versatile and a must read by all in future. This demands high-quality original articles from all young scientists, nurses, social workers, psychologists, clinicians and caregivers in this noble area of palliative care.

The cover page and contents have undergone a subtle change and I wish to work for further improving the quality. This issue onwards we have made this journal fully online including submission, review, review corrections etc. Eventually, I wish the number of issues to increase from biennial to quarterly depending on the popularity and increased awareness of palliative care in the countries of the developing world.

In the end, I would like to thank all the contributors of original as well as review articles and case reports in this issue. In future, I look forward to support from all spheres to build quality awareness in this small but upcoming society of people concerned with the quality of life of patients requiring palliative care.